# Contamination Profile and Health Status of *Mya arenaria* in the Saguenay–St-Lawrence Marine Park

**DOI:** 10.3390/jox16060201

**Published:** 2026-10-20

**Authors:** Francois Gagne, Chantale André, Samuel Turgeon, Nadia Menard

**Affiliations:** 1 Environment and Climate Change Canada, 105 McGill, Montréal, QC H2Y 2E7, Canada; chantale.andre@ec.gc.ca; 2 Parks Canada, Saguenay–St. Lawrence Marine Park, 182, Rue de l’Église, Tadoussac, QC G0T 2A0, Canada; samuel.turgeon@pc.gc.ca (S.T.); nadia.menard@pc.gc.ca (N.M.)

**Keywords:** *Mya arenaria*, plastic contamination, metals, oxidative stress, protein aggregation, shell growth, carbonic anhydrase

## Abstract

Clams represent an important part of the marine benthic community and are food sources for invertebrates (worms, amphipods), fish, and mammals. The purpose of this study was to examine the contamination and health status of feral *Mya arenaria* clam populations from the Saint-Lawrence river and Saguenay fjord at sites of anthropogenic pollution (harbors), a high residency beluga whale area and a reference site under no direct contact of pollution. Intertidal clams were collected for nanoplastics (NPs), dibutyl phthalate (DBP), metal contamination and health status assessments. Health status consisted of the evaluation of oxidative stress, protein aggregation, esterase, and carbonic anhydrase at the biochemical level, followed by morphological assessments (shell mass, condition factor, growth index) and resistance to air emersion. The data revealed that clams collected from polluted sites had elevated levels of NPs, DBP, Cd, Ce, Fe, Cu, Sn and Zn compared to the reference site. Surprisingly, the beluga high residency area site was also contaminated by these contaminants (with the exception of Cd), especially for Sn, NPs and DBP. These clams contained elevated levels of peroxidase activity, protein aggregation, esterase, (mantle) carbonic anhydrase and accumulated lipids. The clams displayed at least at one polluted or whale residency site, decreased condition factor, shell mass ratio, air survival time and increased growth index. Cluster analysis revealed that the above contaminants were strongly associated with carbonic anhydrase, esterase, peroxidase and protein aggregation changes but less so with morphological changes. In conclusion, feral clam populations are contaminated by plastic compounds, including the plasticizer DBP and Sn, contributing to reduced shell mass.

## 1. Introduction

Marine and freshwater clams are important members of the benthic community, serving as a food source for many species (worms, fish and mammals) and contributing also to CO_2_ fixation for shell formation and growth [1,2]. The soft clam *Mya arenaria* thrives in less compact sediments (3–10 cm deep) and interacts closely with the water column for breathing and feeding on suspended particles. The clams are found at intertidal mudflats and subtidal sediments across the eastern and western/northern coast of North America. They contribute to sediment mixing and oxygenation and under predation pressures from birds, worms (*Nereis* sp.), fish and marine mammals, including beluga whales. Stomach content analysis revealed that these whales feed on *Nereis* sp., in addition to shrimps and fish and to a lesser extent bivalves, depending on the compactness (sandy) of sediments [3,4,5]. *Mya arenaria* clams have also served as a traditional food for coastal communities over the last centuries, hence are a potential pathway of contamination for the human population as well.

Clams are sexually dimorphic, reaching sexual maturity at shell lengths of >2.5 cm for this species [6]. They reproduce by external fertilization during early summer at the end of June/early July. Spawning usually follows increases in water temperature (12–20 °C). Other factors include food availability and quality, which can be compromised by the occurrence of pollutants and toxins. Morphological changes are non-destructive methods such as the condition factor (clam total weight/shell length) and age evaluation (major grooves on shells). A recent review revealed that physiological targets affected by pollution (DNA damage, oxidative stress, metal-binding proteins, altered gametogenesis, reduced immunocompetence) were statistically related to three simple morphological biomarkers requiring no special laboratory equipment [7]. These were the “condition” factor, the growth index and air survival time. The air survival test, also coined as the stress-on-stress (SOS) test, determines the capacity of individuals to withstand air emersion until death, which integrates the capacity to endure underlying (and cumulative) stresses such as disease, pollution, malnutrition, anoxia and impacts of pollution. These studies revealed that contaminant-induced damage ultimately scales up to bivalve morphology and resistance to stress (air emersion). In these times of global warming, bivalves could be exposed to longer periods of drought and high temperatures in already contaminated environments either in freshwater or marine ecosystems with dire consequences. The occurrence of plastic nanoparticles (NPs) in addition to known organic and inorganic contaminants is a growing concern of the scientific community as plastics are found in virtually every compartment of aquatic and soil ecosystems [8]. Moreover, the co-occurrence of plastic nanoparticles was shown to potentiate and increase the toxicity of other contaminants, for example, with cadmium and polystyrene nanoparticles [9].

The purpose of this study was to examine the relationships between the NPs, a common plasticizer dibutyl phthalate (DBP), selected elements/metals and the health status of intertidal clams in the Saguenay–St. Lawrence Marine Park. The survey was conducted to seek out spatial (intersite) differences and was sampled at one time well after the spawning period to alleviate reproduction-related effects. Health status was examined by following changes in CF, GI and air survival, as proposed by Blaise et al. [7], followed by the evaluation of impacts aimed to underly other stresses on protein stability, shell regeneration and plastic ester biotransformation, such as protein condensation, carbonic anhydrase in the mantle where shell growth occurs and carboxylesterase activity respectively. Special attention was made to underly potential relationships between NPs, tin and copper contamination and shell integrity. As a corollary, since clams are the main food source for the worms on which belugas feed, the protection of its prey and habitat is mandatory for the recovery of beluga whale populations and endangered marine mammals.

## 2. Materials and Methods

### 2.1. Study Sites and Clam Collection

**Figure 1:**
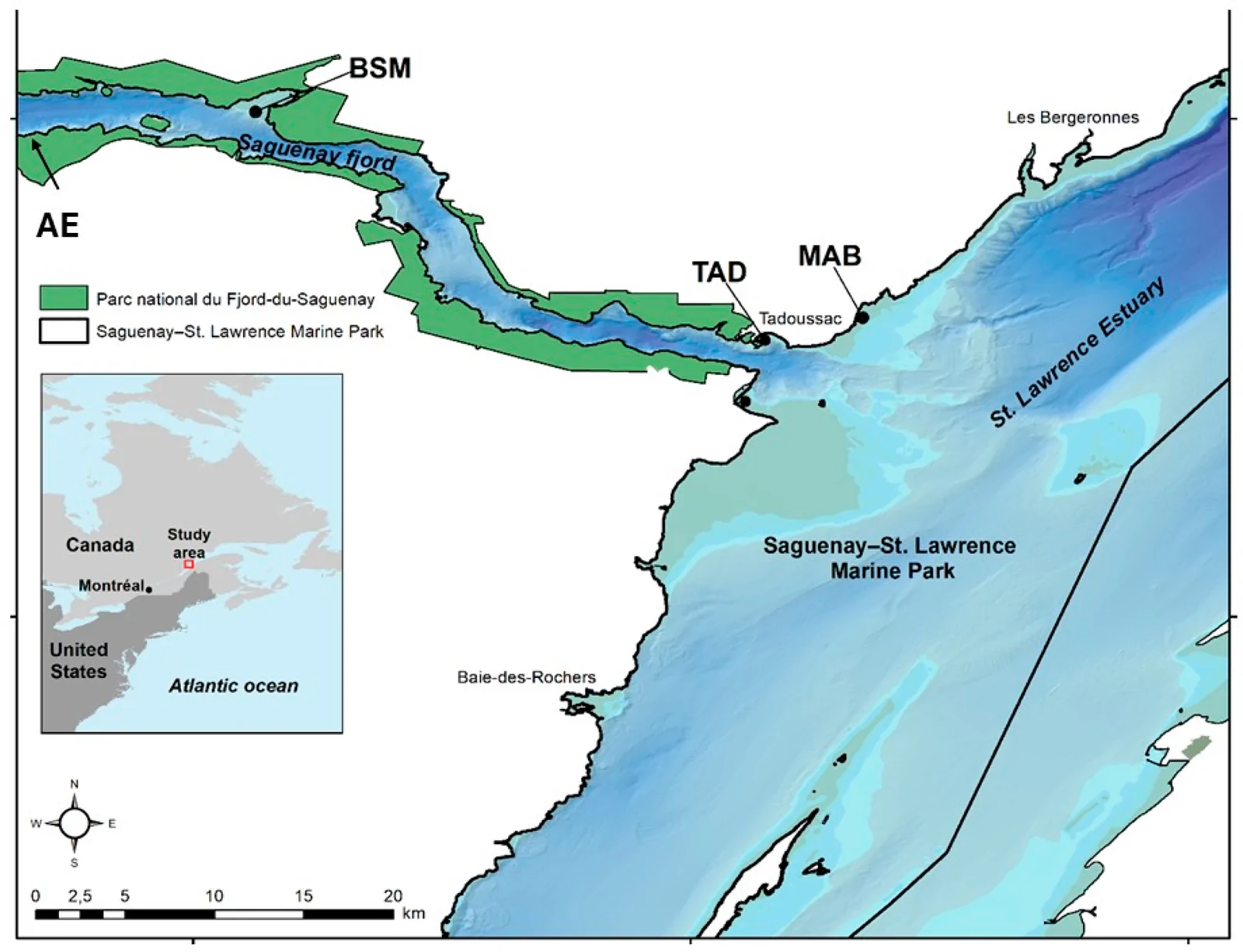
Study area. The location of four *Mya arenaria* intertidal sampling sites: TAD (Baie de Tadoussac), a site subject to wharf, marina and wastewater discharge pollution; MAB (Baie du Moulin-à-Baude), a reference site located upstream of the Baude river; AE (Anse aux Érables), a site located upstream of the fjord receiving the input from large cities upstream of Saguenay Fjord (Saguenay city); BSM (Baie Sainte-Marguerite), a beluga whale high residency area.

In total, 40 *Mya arenaria* clams (shell length > 5 cm) were randomly selected at each of the 4 sites in the last week of September 2023 in the St. Lawrence-Saguenay Marine Park sites, as depicted (Figure 1). The reference site was Baie du Moulin à Baude (MAB), sampled in mudflats located 1 km upstream of the outfall of Rivière du Moulin à Baude. This site was located upstream of the River Baude and was not under direct contact with sources of pollution. The lower levels of contamination in clam tissues were found in previous studies over the last 25 years [10]. The polluted sites were Anse aux Érables (AE) and Tadoussac (TAD). These are close to recreational and commercial boating activities (TAD) and downstream of relatively large cities and two commercial ports and marinas upstream in the Saguenay Fjord (AE). The last site consists of a high residency beluga whale area located in the Baie Sainte-Marguerite (BSM). This site is frequented by females and young during the summer for feeding [11,12]. Although this site was not directly exposed to sources of pollution, recent studies revealed contamination problems from nearby boats and the perhaps domestic and industrial pollutants released from the Sainte Marguerite River [13,14].

The clams were placed in coolers filled with seawater and transported live to the Parks Canada laboratory at Tadoussac and allowed to depurate at 4 °C for 3–4 h. Wet weight, longitudinal length and age (number of major grooves on shells) were measured. A subset of 15 clams was randomly sampled for air survival time evaluation [7,13] and the remainder were stored at −85 °C. For air survival, the clams were placed in 80% humidity at 15 °C and weighted each day until shell opening (death). The lethal time (LT in days) and dehydration rate (DHR: weight at time of death-initial weight/initial weight) were also determined.

For subsequent chemical and biomarker analyses, the clams were thawed at 4 °C on ice and the shells were separated from the soft tissues and mantle tissues were weighed to determine the shell mass ratio (SMR: shell weight/total clam weight), condition factor (CF: clam weight/shell length), and growth index (GI: shell length/age). The tissues were then homogenized as described below.

### 2.2. Contaminants Analysis in Clams

Elemental analysis of clam tissues was performed to determine the levels of selected contaminants such as Al, Ca, Cd, Ce, Cu, Fe, K, Sn and Zn that were previously identified as key elements affected in this area [13]. Other contaminants also include dibutyl phthalate (DBP) and nanoplastics (NPs) in soft tissues. For elemental analysis, soft tissues from *n* = 6 individuals were separately homogenized using a Polytron tissue grinder (Fisher Scientific, Ottawa, ON, Canada) nd 1 mL was acid digested in 4 mL of 8 M HNO_3_ at 240 °C under pressurized vessels in a microwave oven. The samples were then diluted to 50 mL with 1% HCl before analysis using ion coupled plasma quadrupole mass spectrometry (ICP-MS, XSERIES 2 from Thermo Scientific, Waltham, MA, USA) [15]. The data were expressed as µg/g wet weight. For the plastic compounds DBP and NPs, a 0.3 mL sample of soft tissues homogenate was extracted in saturated NaCl (5 M) and acetonitrile (ACN), mixed for 10 min, and centrifuged at 150× *g* for 5 min to separate the ACN phase. The levels of NPs in the ACN fraction were determined using gold nanosensor (nAu) methodology, as previously described [16]. Uncoated gold nanoparticles (10 nm diameter; 25 mg/mL from Nanocomposix, San Diego, CA, USA) were coated with 0.01% mercaptoundecanoic acid (MUA) for 12 h at 4 °C, centrifuged at 20 000× *g* for 10 min to remove free MUA and the pellet (nAu-MUA) resuspended in MilliQ water. A 10 µL volume of the ACN extract was mixed with 120 µL of nAu-MUA suspension followed by the addition of an aggregation agent (10 mM HCl), and the aggregation ratio was determined at 620 nm/530 nm, which decreased upon binding NPs. A standard solution of polystyrene nanoparticles in the ACN extract was used for calibration (20 nm diameter, Thermofisher Inc., Waltham, MA, USA). The detection limit was 50 ng of 50 nm polystyrene nanoparticles/mg tissues. The data were expressed as ng polystyrene equivalent/mg tissues. The determination of DBP in tissues was determined using an arginine-coated nAu sensor, as previously described [17]. The principle of the assay is based on the properties of positively charged arginine-coated nAu, which keeps them from aggregating but, upon binding DBP to the nAu, leads to a reduction in the electrostatic repulsion between the nanoparticles, where aggregation is facilitated following the addition of diluted HCl. First, citrate-coated 10 nm nAu nanoparticles at 25 mg/mL were purchased from Nanocomposix (San Diego, CA, USA) and centrifuged at 20,000× *g* for 10 min at 4 °C to remove excess citrate from the pelleted nAu. The pellet was resuspended in 0.001% arginine for 3 h at room temperature. Another centrifugation step (similar speed) to remove excess and unbound arginine was performed. The assay consisted of mixing 120 µL of arginine–nAu with 10 µL of the ACN fraction and 10 µL of HCl 0.1 M. The absorbance was then measured between 500 and 750 nm to determine changes between dissociated nAu (A 530 nm) and aggregated nAu (A 620 nm), which was increased for the latter in the presence of DBP. Calibration was achieved using DBP from (1–10 µg/mL). The data were expressed as µg DBP/g tissues.

### 2.3. Biomarker Analysis

Tissue homogenates were prepared from *n* = 8 clams in 140 mM NaCl containing 5 mM KH_2_PO_4_ and 1 mM EDTA, pH 7.4 using a Teflon pestle tissue grinder (Fisher Scientific, On, Canada) on ice. Operational blanks (MilliQ water only) for elemental analysis were added to tease potential contamination from the polytron rod. The homogenate was then centrifuged at 3000× *g* for 10 min at 2 °C. The supernatant (S3 fraction) was collected from the pellet and stored at −85 °C. The levels of total proteins were determined using the 280 nm absorbance method in the presence of 10 mM NaOH, 1 mM EDTA and 0.1% SDS. A standard solution of serum albumin was used for calibration. The data were expressed as mg/mL total proteins. Total lipids were determined using Nile red dye, as previously described [18]. Briefly, the S3 fraction of the homogenate was mixed with 10 µM Nile red in phosphate buffered saline (140 mM NaCl, 5 mM KH_2_PO_4_, pH 7.4) and fluorescence was measured at 485 nm excitation and 560 nm emission. Calibration was achieved with canola oil dissolved in ethanol. The data were expressed as relative fluorescence units (RFUs)/mg total proteins in the S3 fraction.

Protein aggregation (Prot agg) was determined using thioflavine T fluorescence probe methodology [19]. Briefly, a 50 µL sample of the S3 fraction of homogenates was mixed with 150 µL of 100 µM thioflavine T in phosphate buffered saline for 10 min. Fluorescence was measured at 440 nm excitation and 485 nm emission in a dark microplate (Synergy-IV, Biotek Instruments, Winooski, VT, USA). The data were expressed as relative fluorescence units (RFUs)/mg proteins. Peroxidase was measured using the fluorescent substrate dichlorofluorescein and H_2_O_2_, as previously described [20]. Briefly, 20 µL of the S3 fraction was mixed in 180 µL of 10 µM of 2,7-dichlorofluorescein and 0.01% H_2_O_2_ for 20 min at 20 °C. Readings at 485 nm excitation/530 nm emission were taken each 4 min in dark microplates (Synergy-IV, Biotek instruments, Vermont, USA). The data were expressed as the increase in fluorescence/min and normalized to total proteins. Carboxylesterase activity was also determined in the S3 fraction of soft tissues using the spectrophotometric assay using fluorescein diacetate (FDA). Briefly, 20 µL of S3 fraction were mixed with 180 µL of 100 uM FDA in PBS buffer. The increase in fluorescence was measured at 485 nm excitation and 530 nm emission at each 5 min for 20 min at 20 °C in clear microplates (Synergy IV, Biotek Instruments Vermont, Winooski, VT, USA). The data were expressed as relative fluorescence unit (RFU)/min normalized with total proteins. Carbonic anhydrase (CA) activity was determined using p-nitrophenyl acetate [21]; the assay was performed on the mantle tissues and corrected against total lipids to control for non-specific esterase (lipase) activity using covariance analysis (lipids as covariate).

### 2.4. Data Analysis

Clams (15, 8 and 6 for morphological, biomarker and chemical analysis, respectively) were collected at two polluted sites (TAD, EA), one high beluga residency site suspected to suffer from contamination problems (BSM) and one reference site (MAB) in the St. Lawrence Estuary and Saguenay Fjord. The data were subjected to an analysis of variance (ANOVA) followed by Tukey’s test, using the MAB site as the reference site. Data normality and homogeneity of variance was performed using the Shapiro–Wilk and Bartlett tests respectively. In the case of any deviance from normality of heterogenous variance, the data were log-transformed before analysis. Correlation analysis of biomarker data (encompassing all treatments) was also performed using the Pearson moment procedure. Cluster analysis using hierarchical tree analysis and relative distance computed by Pearson correlation (1-r) was also performed to seek out relationships between biomarkers of exposure and effects. Significance was set at *p* < 0.05 and the SYSTAT software package was used (version 13, SanJose, CA, USA).

## 3. Results

Clams were collected at four sites depicted in Figure 1 following an upstream/downstream approach in the Saguenay Fjord: AE (salinity = 8–12 g/L), BSM (salinity = 12–18 g/L), TAD (salinity = 18–28 g/L) and MAB (salinity = 20–30 g/L). The morphological features of the clams and the air survival time by sites are presented in Table 1. The CF and SMR of clams were significantly lower at the BSM site compared with the reference site MAB. The GI was significantly higher at the TAD site compared with the reference site MAB. The LT was significantly lower at the sites near anthropogenic activities (AE and TAD) compared with the site MAB, while no significant changes were found for BSM. The DHR did not change across the sites, with the exception at the TAD site with a higher weight loss at time of death. The age of clams was significantly higher at AE, suggesting a low recruitment of younger clams in this area of low salinity. Correlation analysis revealed that CF was significantly correlated with age (r = 0.53) and LT was correlated with GI (r = 0.48). This suggests that older clams have higher weight/shell length ratio and clams that are less resistant to air exposure are those showing low GI.

Contaminant loadings in clam tissues were examined at these sites by measuring the levels of NPs, DBP (Table 2) and the following elements: Al, Ca, Cd, Ce, Cu, Fe, K, Sn and Zn (Table 3). The levels of NPs and DBPwere significantly elevated at the TAD and BSM sites compared with the reference site MAB. The levels of NPs and DBP were significantly correlated (r = 0.9) as expected since DBP is an intrinsic component of plastics. The levels of lipids were also determined because plastics are thought to interact/associate with lipids in tissues [22]. Lipids were elevated at all sites (AE, BSM, TAD) compared with the reference site MAB. Correlation analysis confirmed a significant association between NPs/DBP and lipids (r = 0.65 and 0.62 respectively). The levels of lipids were also correlated with LT (r = 0.44). In the attempt to determine whether increases in NPs and DBP were site-specific or owing to increased lipids, an analysis of covariance was performed with lipids as the covariate. The analysis revealed that changes in NP and DBP levels were mainly driven between sites (*p* < 0.05) rather than lipid levels (*p* > 0.05) in tissues. Zn, Fe, Ce and Sn were significantly elevated at TAD and BSM compared with the reference site MAB. Cu was elevated at all sites while Al levels were increased at the BSM and AE sites. Only Cd was significantly increased at the uppermost site AE. Ca levels were significantly reduced at the BSM site. No significant changes in K levels were observed. The levels of K can be used as markers of toxicity. Indeed, tissue K levels are highly regulated by Na/K ATPase, which keeps high levels of K in tissues/cells relative to sea water reported to contain 400 µg/g corresponding to circa half of the K levels in tissues. Alteration of K homeostasis would lead to decreasing tissue levels of K down to equilibrium with sea K levels.

Correlation analysis revealed that Al was significantly correlated with NPs (r = 0.83), DBP (r = 0.88) and lipids (r = 0.42). The levels of Cd were significantly correlated with LT (r = 0.69) and total lipids (r = 0.42). Ce tissue levels were significantly correlated with NPs (r = 0.83). Cu in tissues whose levels were increased at all sites were significantly correlated with NPs (r = 0.79), DBP (r = 0.78) and lipids (r = 0.83). Total Fe in tissues were significantly correlated with NPs (r = 0.9), lipids (r = 0.54), and DBP (r = 0.9). The levels of Sn (a marker for organotin contamination) in tissues were significantly correlated with NPs (r = 0.67), DBP (r = 0.75) and lipids (r = 0.41).

Finally, Zn was significantly correlated with CF (r = −0.42), NPs (r = 0.8) and DBP (r = −0.89). Taken together, it appears that plastic contamination (NPs and DBP) follows contamination in the following elements: Zn, Sn, Fe, Cu, Ce and Al.

Clam health status was further studied by measuring changes in biomarkers such as peroxidase (Perox), protein aggregation (Prot Agg), esterase and CA activities (Table 4). The activity of Perox was significantly induced at the anthropic site TAD and the beluga high residency site BSM. Perox activity was significantly correlated with NPs (r = 0.87), lipids (r = 0.63), DBP (r = 0.92), Zn (r = 0.85), Al (r = 0.83), Cu (r = 0.8), Sn (r = 0.61), Ce (r = 0.84) and Fe (r = 0.9). Protein aggregation (amyloids) was significantly increased at BSM only. Correlation analysis revealed that Prot Agg were correlated with NPs (r = 0.87), lipids (0.5), DBP (r = 0.9), Zn (r = 0.91), Al (r = 0.94), Cu (r = 0.78), Sn (r = 0.76), Ce (r = 0.96) and Fe (r = 0.89). Esterase activity was significantly increased at all sites (AE, BSM and TAD). Esterase activity was significantly correlated with NPs (r = 0.91), DBP (r = 0.95) and lipids (r = 0.58), Zn (r = 0.82), Al (r = 0.73), Cu (r = 0.78), Sn (r = 0.73), Ce (r = 0.86) and Fe (r = 0.85). Carbonic anhydrase (CA) activity in the mantle involved in the fixation of carbonate during shell production was significantly higher at the BSM site. Correlation analysis revealed that CA was significantly correlated with Ca (r = −0.58) CF (r = −0.54), NPs (r = 0.49), DBP (r = 0.68), Zn (r = 0.76), Al (r = 0.51) Cu (r = 0.5), Sn (r = 0.57), Ce (r = 0.76) and Fe (r = 0.51).

**Figure 2:**
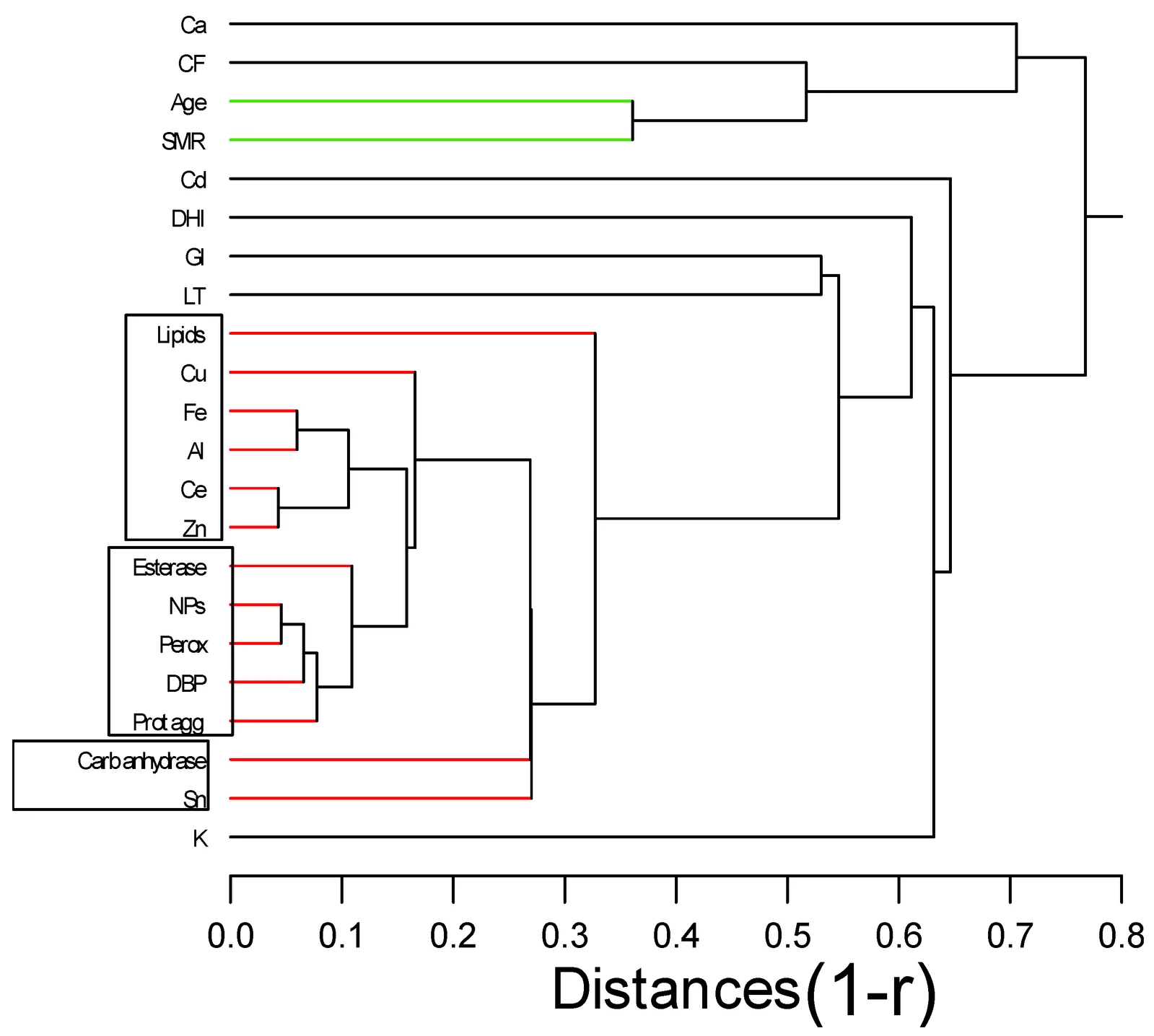
Hierarchical tree analysis of biomarker responses in clams. Hierarchical tree analysis was performed using (1-r) as the measure of distance between biomarkers. In this analysis, biomarkers of exposure (NPs, DBP, Cd, Ce, Cu, Fe, Sn and Zn), biomarkers of effects (K, Perox, protein aggregation, esterase, lipids and CA) and health status (CF, GI, LT and age) were considered. The red and green lines indicate significant trends between these endpoints. The boxes are considered significant groups or clusters of the exposure, effects and health status biomarkers.

In the attempt to gain a global perspective clam contamination and health status profile, a cluster analysis using a hierarchical tree analysis was performed (Figure 2). The relative distance between biomarkers was extracted using the 1-Pearson correlation rule. We also performed the analysis using the non-parametric rule (1-Gamma correlation), which gave similar results. The analysis identified three distinct clusters based on the exposure, effects and morphological (health) status of clams. The first cluster comprises Sn and CA activity involved in the fixation of CO_2_ for pH regulation and shell formation. The second cluster includes protein aggregation, Perox, esterases, DBP and NPs. The last cluster includes metals Cu, Ce, Al, Fe and Zn. This cluster was closely linked to the second cluster, suggesting that these contaminants might explain in part the observed effects. Changes at the morphological level (CF, GI, LT, Age) and K homeostasis were less related to the three clusters described above, suggesting that these changes have yet resonated/scaled at this level; i.e., clams are under stress but not at the morphological/global health level.

## 4. Discussion

Based on the contamination profiles, the TAD and BSM sites were the most impacted in NPs, DBP, Ce, Fe, Cu and Sn, although with relatively small replication between sites (*n* = 6 individuals per site). The assay for NPs was previously shown to respond not only to polystyrene (used for calibration) but to other types of plastics [16], hence is the reported levels are not solely based on the absolute levels of polystyrene. Indeed, the assay was able to detect different polystyrene sized (10–50 nm) up to 100 nm [23], polyethylene (aged bottles) and tire wear particles. The site AE, located upstream of the Saguenay Fjord but downstream of relatively large cities and commercial ports, had elevated levels of Al, Cd and Cu. The salinity of this upstream site was somewhat lower than the others and might have contributed to increase the bioavailability of some of the measured elements. These responses were obtained at a single point during the year corresponding to a lag phase period of reproduction (October) for this species. This area was reported to support several aluminum and pulp and paper industries [10]. Tissue levels of Sn and Cu (and NPs and DBP) found at some sites supporting the use of antifouling paints from industrial activities or commercial boating activities (BSM and TAD but not AE). This suggests the presence of antifouling plastic and Sn/Cu-containing paints currently used in large cargo boats or from other industrial paints [24,25,26]. The occurrence of Sn in tissues was previously reported at the BSM site, suggesting that this contamination problem persistshas been over the years [13]. Future studies should examine more closely the contribution of sediment and surface waters to the reported levels of elements in clam tissues in the attempt to identify environmental sinks for these compounds. Moreover, Sn tissue levels were significantly correlated with CA activity (r = 0.58), for the fixation of calcium carbonates to support shell growth but with reduced SMR at BSM where high Sn levels were observed. This suggests that shell mass reduction occurs downstream of the CA step, perhaps at the fixation of calcium to the mantle proteins. Mantle proteins are associated with chitin precursors to ensure calcium (whose levels were reduced at BSM) and carbonate fixation [27]. It is possible that Sn and NPs could disrupt these processes by competing/binding Ca to these shell proteins. This hypothesis should be confirmed by more specific experiments. Reduced growth in mussels and clams was reported at sites contaminated with high TBT levels ranging from 0.3 to 0.9 µg/L in seawater [28]. The estimated bioconcentration factor of TBT from the octanol/water coefficient (log K_ow_ 3.54) was estimated at 110, giving whole tissue levels in TBT in the range of 33–100 ng/g. This is in the same range of the mean Sn levels in tissues (70 ng/g) at BSM in the present study. Exposure to organotin compounds was also found to increase shell thickening in oysters, but shell mass does not necessarily follow [29]. A weak correlation (r = 0.37), albeit significant between shell thickness and shell weight, was reported in blue mussels [30], suggesting that shell thickness should be somewhat related to shell mass. However, this should be confirmed more closely in future investigations in *Mya arenaria* clams. Clams exposed to organotin (0.15 µg/L) during feeding grew less and displayed abnormal shell growth but with no change in shell length. Following 56 days of exposure treatment, shell thickening occurred at Sn tissue levels of 0.59 µg/g, which was substantially higher than the levels found in the present study (upper range of 0.07–0.18 µg/g). This corroborates the decrease in SMR in clams from the BSM site in the present study, although increased CA was uncoupled with shell weight. The data also revealed that, at 0.09 µg Sn/g, no significant changes in shell weight and thickening were observed in oysters. CA is a zinc-containing enzyme and Zn levels were readily increased at the BSM site (at concentrations 150-fold higher than Sn in tissues), suggesting that the increase in CA activity was perhaps more strongly influenced by Zn than Sn in tissues. It is possible that high Zn levels promote or protect CA activity against other metals (inhibitors); however, no evidence was found in the literature that Zn could induce CA gene expression and translate into an active enzyme, albeit the enzyme requires Zn for functioning. Moreover, there is no clear evidence that organic/ionic Sn either inhibits or activates CA activity. It was reported that TBT could decrease the H^+^ gradient by Cl-/OH- exchanges, which neutralizes H^+^ in cells [31]. This could increase CA activity by favoring HCO_3_^−^ ⟶ CO_3_^−2^ + H^+^, given that the pKa of HCO_3_^−^ is 10.2. On the other hand, triethyltin was a mild inhibitor of CA, reaching only a 10–15% decrease in activity with 0.1–2 mM triethyltin [32]. It is possible that increased CA activity was a compensation mechanism to maintain HCO_3_^−^ levels for clam shell growth and pH maintenance, especially with the abundance of Zn in tissues. CA activity could also be induced by hypoxia [33] and low salinity [34], although the relationship with Zn in these conditions is not clear. It is well known that Sn could bind and inactivate heme-containing proteins (myoglobin), which could alter heme metabolism and perhaps contribute to hypoxia [35]. More research should address this to better understand the long-term health effects of Sn pollution in bivalves in respect to shell status.

Clams contained high levels of Al and Fe, consistent with bauxite extraction and deposits from the aluminum industry in this region i.e. near AE and BSM. Bauxite contains 50% of Al and 10–25% Fe by weight [36], giving an Al/Fe ratio of 2–5. Industrial Al extraction removes between 60 and 80% of Al, and Fe is not captured. This gives the Al/Fe ratio of 0.4 in residual bauxite (after Al extraction) that could be released in the aquatic environment. At the BSM site, the Al/Fe tissue ratio was 0.33, somewhat like the Al/Fe ratio of residual bauxite. Al and Fe were closely associated with Perox and esterase activities, suggesting an enhanced capacity of tissues to remove reactive oxygen species and esters including fatty acids (lipids), which were increased at the anthropic sites AE, BSM and TAD. It is well known that Fe is involved in the production of hydroxyl radicals from H_2_O_2_ during the Fenton reaction [37]. Al was shown to have pro-oxidant activity by reducing Fe(III) to Fe(II), which promotes the formation of hydroxyl radicals from H_2_O_2_ [38]. This is consistent with the observed increase in Perox activity at sites contaminated by Al and Fe and consists perhaps of a protective mechanism/response against oxidative stress.

It was noteworthy that NPs were detected in clam tissues at the anthropic sites AE and BSM. This was paralleled with tissue levels of DBP, a well-known plasticizer. Moreover, NPs and DBP were significantly correlated with tissue Sn at r = 0.67 and r = 0.75 respectively. This suggests that these compounds could originate from marine paints, known to contain plastics, DBP [39] and Sn (US6710117B2-Marine paint compositions-Google Patents) [40]. Plastics and other contaminants from paints are often overlooked as a source of plastics in the marine environment. Indeedm micro/nanoplastics and DBP are often correlated with each other given they are found in the same paint base [8], indicating that plastic materials are part of a more complex mixture in the environment. Moreover, paint micro/nanoparticles are regularly detected in sediments and the digestive system of resident animals. They also contain paint additives contributing to toxicity such as Cu^+^/Cu^2+^, organotin, Pb^2+^ and CrO_4_^2−^ within the paint matrix. NPs are closely associated with peroxidase and protein aggregation, suggesting the formation of protein aggregation, a precursor of protein plaques (amyloids) if this stress is maintained for the long-term. Protein plaques are often observed in many degenerative diseases and aging such as atherosclerosis and neural impairment (Alzheimer’s, Parkinson’s, and Huntington’s diseases). Plastic particles are shown to induce oxidative stress and stimulate protein plaques from steric hindrance/crowding, leading to protein misfolding [41,42]. This also supports previous findings in protein aggregation and NPs in *Mya arenaria* clams collected at the same TAD site [43]. This suggests that the onset of degenerative disease from the accumulation of protein plaques, however, was not confirmed in the present study. Clams sampled at the BSM site were the youngest (mean age of 7.5 years compared with 8.2 and 8.3 years for the MAB and TAD site respectively), but this could be the result of predation (worms, birds, beluga and other mammals), although we did not observe any changes in empty shells (dead clams) on the sand banks. A threshold range of clam age could be established in the present study by calculating the mean and 95% confidence interval of clam age across all sites: 8.5 years (95% CI: 8.1–8.8) where values below 8.1 years could be considered a significant drop in clam age (as observed at BSM site), although this does not confirm early clam death from degenerative diseases. This parameter could serve as an early warning test (alarm) to seek out the onset ofdegenerative diseases, albeit not a definitive test since other factors should be considered (predation, diseases). Based on the data presented in the present study, the weight of evidence corroborates the notion that clams at the Sn and plastic contaminated site BSM are younger than those at the other sites and show lower shell mass.

## 5. Conclusions

Local feral clams were contaminated by plastic compounds (DBP and NPs) and selected elements (Al, Ce, Fe, Cu and Sn) and displayed various signs of stress at the physiological level such as peroxidase, protein aggregation, carboxylesterase and CA. Changes were also observed at the organism levels (CF, GI, LT and SMR), but cluster analysis revealed that they were less related to physiological markers and plastic/elemental contamination. This suggests that the contamination status of clams does not directly harm the organisms, although they are suffering from stresses at lower levels of biological organization. It is noteworthy that clam age and shell mass were significantly lower at the BSM site. and The clams were contaminated the most by Sn, DBP and NPs.

## Figures and Tables

**Table 1:** Morphological characteristics of clams.

Sites	CF (g/mm)	GI (mm/Years)	Shell Weight ^a^	LT (Days)	DHR (%)	Age (Years)
MAB	0.53 ± 0.03	8.1 ± 0.2	0.602 ± 0.004	9.8 ± 2	31 ± 2	8.2 ± 0.3
AE	0.56 ± 0.06	7.6 ± 0.4	0.614 ± 0.003	4.5 ± 1 *	34 ± 4	9.6 ± 0.3 *
TAD	0.47 ± 0.03	9.6 ± 0.3 *	0.605 ± 0.004	6.6 ± 2	42 ± 2 *	8.3 ± 0.3
BSM	0.35 ± 0.02 *	8.0 ±0.2	0.570 ± 0.007 *	8.3 ± 1	32 ± 1	7.5 ± 0.3 *

The data represent the mean and standard error. The star symbol * indicates significant difference relative to the reference site MAB; ^a^ shell weight ratio (g shells/g clam).

**Table 2:** Occurrence of nanoplastics and DBP in soft tissues of *Mya arenaria* clams.

Sites	NPs µg/g Tissues	DBP µg/g Tissues	Lipids mg/g Tissues
MAB	0.06 ± 0.006	1.7 ± 0.003	2.1 ± 0.6
AE	0.06 ± 0.005	1.65 ± 0.003	4.1 ± 0.8 *
TAD	0.11 ± 0.009 *	2.9 ± 0.01 *	3.7 ± 0.5 *
BSM	0.14 ± 0.008 *	4.4 ± 0.009 *	5.8 ± 0.9 *

The data represent the mean and standard error. The star symbol * indicates significant difference relative to the reference site MAB.

**Table 3:** Inorganic elements in tissues.

	MAB	TAD	BSM	AE
Elements (µg/g)				
Al	84 ± 3	82 ± 8	253 ± 20 *	45 ± 5 *
Ca	1485 ± 224	1427 ± 100	696 ± 40 *	1643 ± 125
Cd	0.10 ± 0.005	0.09 ± 0.01	0.12 ± 0.01	0.13 ± 0.008 *
Ce	0.4 ±0.02	0.7 ± 0.05 *	2.2 ± 0.1 *	0.4 ± 0.06
Cu	0.6 ± 0.2	1.2 ± 0.1 *	2.3 ± 0.2 *	1.3 ± 0.2 *
Fe	210 ± 20	415 ± 31 *	767 ± 75 *	210 ± 5
K	858 ± 23	905 ± 52	893 ±43	858 ± 25
Sn	0.01 ± 0.004	0.02 ± 0.004 *	0.07 ± 0.02 *	0.007 ± 0.001
Zn	8.4 ± 0.3	11 ± 0.7 *	17 ± 0.8 *	8.3 ± 0.1

Sea water equivalent: 400–420 µg/g of K. The star symbol * indicates significance from the reference site BMB.

**Table 4:** Biomarkers of toxicity in *Mya arenaria* clams.

Sites	Peroxidase	Protein Aggregation	Esterase	Carbonic Anhydrase (CA)
MAB (reference)	7 ± 0.8	65 ± 4	0.6 ± 0.04	1 ± 0.15
TAD	13 ± 1 *	71 ± 3	1.3 ± 0.1 *	1.2 ± 0.3
BSM	18 ± 1 *	151 ± 6 *	1.7 ± 0.2 *	2.2 ± 0.5 *
AE	8 ± 0.4	48 ± 3	0.9 ± 0.03 *	0.9 ± 0.4

The star symbol * indicates significance from the reference site BMB.

## Data Availability

The raw data supporting the conclusions of this article will be made available by the authors on request.
